# TaqMan-probe-based multiplex real-time qPCR assay for simultaneous detection of Duck Tembusu virus, Novel duck reovirus, and Duck circovirus

**DOI:** 10.3389/fmicb.2026.1884322

**Published:** 2026-07-21

**Authors:** Zhixian Wang, Haoran Xi, Jia Tan, Cui Lin, Xiaona Gao, Siyi Wang, Haiqin Li, Xiaoquan Guo

**Affiliations:** 1 Jiangxi Provincial Key Laboratory for Animal Health, College of Animal Science and Technology, Jiangxi Agricultural University, Nanchang, China; 2 Institute of Animal Husbandry and Veterinary Medicine, Jiangxi Academy of Agricultural Sciences, Nanchang, China; 3 Jiangxi Provincial Key Laboratory of Green and Healthy Breeding of Livestock and Poultry, Nanchang, Jiangxi, China

**Keywords:** Duck circovirus, Duck Tembusu virus, multiplex real-time qPCR, Novel duck reovirus, TaqMan

## Abstract

Duck Tembusu virus (DTMUV), Novel duck reovirus (NDRV), and Duck circovirus (DuCV) are the primary epidemic pathogens threatening China’s waterfowl farming industry. The immunosuppressive characteristics of these viruses readily facilitate mixed infections between pathogens, thereby increasing the difficulty of clinical diagnosis and disease prevention and control. To achieve rapid simultaneous detection of these three pathogens, this study designed specific primers and probes based on conserved viral regions, successfully establishing a TaqMan multiplex real-time quantitative PCR assay. The results demonstrate that the method established in this study exhibits excellent specificity and shows no cross-reactivity with other pathogens in waterfowl. The limit of detection (LOD) of this method for the three pathogens was 10^1^ copies/μL, representing a 100-fold improvement in detection sensitivity compared to conventional PCR assay. Repeatability experiments demonstrate that this method exhibits excellent stability and reproducibility. Utilizing this method, we analyzed 120 clinical samples collected from diseased ducks across different regions of Jiangxi Province, China. Compared with conventional PCR, the detection rates for the three viruses were substantially higher, and co-infections with these viruses were also identified among the samples. In summary, the method established in this study offers distinct advantages of high specificity, sensitivity, and reproducibility. It enables rapid identification of these three pathogens in a single reaction, providing an efficient and viable technical approach for the rapid diagnosis of mixed viral infections in waterfowl farming.

## 1 Introduction

Waterfowl farming is a crucial component of the livestock industry. In recent years, with increasing stocking densities and a growing complexity in disease patterns, polyviral infections among waterfowl have become increasingly prevalent worldwide. This phenomenon has led to a series of issues including high mortality rates and diminished production performance, resulting in significant economic losses for the waterfowl farming industry (Li et al., 2025). In routine waterfowl husbandry, Duck Tembusu virus (DTMUV), Novel duck reovirus (NDRV), and Duck circovirus (DuCV) are prevalent pathogens (Farkas et al., 2025; Li et al., 2025; Qiu et al., 2021). DTMUV belongs to the genus *Flavivirus* within the family *Flaviviridae* and is a single-stranded positive-sense RNA virus (Li et al., 2023). Since its initial outbreak in China in 2010, this virus has posed a serious threat to the country’s waterfowl farming industry (Cao et al., 2011; He et al., 2019; Hu et al., 2020). Following DTMUV infection, affected ducks exhibit reduced appetite, stunted growth, and decreased egg production. Prolonged infection can induce neurological symptoms and even led to death in severe cases (Li et al., 2015; Sun et al., 2019). NDRV belongs to the genus *Reoviridae* within the family *Orthoreovirus*. It was first detected in Fujian Province, China, in 2005, and has since caused outbreaks in multiple regions across the country (Kong et al., 2023; Zhang et al., 2021). The virus primarily affects the immune organs of ducklings, with affected ducks exhibiting irregular hemorrhages and necrotic lesions in tissues such as the liver and spleen (Li et al., 2016; Zhang et al., 2023). DuCV belongs to the genus *Circovirus* within the family *Circoviridae* and is a single-stranded circular DNA virus (Niu et al., 2018; Wang et al., 2022). This virus can infect duck flocks of varying ages, with those under 6 weeks old being the most susceptible. The virus primarily compromises the immune system of ducklings, causing immunosuppression and rendering the flock more susceptible to other pathogens, thereby leading to more severe mixed infections (Cui et al., 2023; Wang et al., 2024).

In waterfowl farming, DTMUV, NDRV, and DuCV are frequently detected viral agents. Meanwhile, these viruses possess immunosuppressive properties, facilitating mixed infections with other viruses and posing a serious threat to the healthy development of the waterfowl farming industry (Yin et al., 2023; Pan et al., 2024). To address this situation, this study designed corresponding primers and probes based on the conserved regions of each virus, successfully establishing a TaqMan multiplex real-time qPCR assay. This method enables rapid and precise identification of the three pathogens within a single reaction, providing a reliable diagnostic tool for clinical diagnosis in the waterfowl farming industry.

## 2 Materials and methods

### 2.1 Clinical samples and viral strains

Unrelated common avian viruses including Duck plague virus (DPV), Newcastle disease virus (NDV), Duck adenovirus type 4 (DAdV-4), Muscovy duck reovirus (MDRV), Goose astrovirus genotype 1 (GoAstV-1), Goose astrovirus genotype 2 (GoAstV-2), Goose parvovirus (GPV), Avian influenza virus (AIV, H5N6), Goose Reovirus (GRV), Fowl adenovirus (FAdV), Duck hepatitis A virus type 3 (DHAV-3), Duck hepatitis A virus type 1 (DHAV-1), Duck Astrovirus (DAstV) were isolated and preserved by our laboratory and were used as unrelated controls. Additionally, during 2024–2025, our laboratory has collected DTMUV, NDRV, and DuCV positive samples. All samples have been confirmed via sequencing and were stored in the laboratory.

### 2.2 Primers and probes

**Figure 1:**
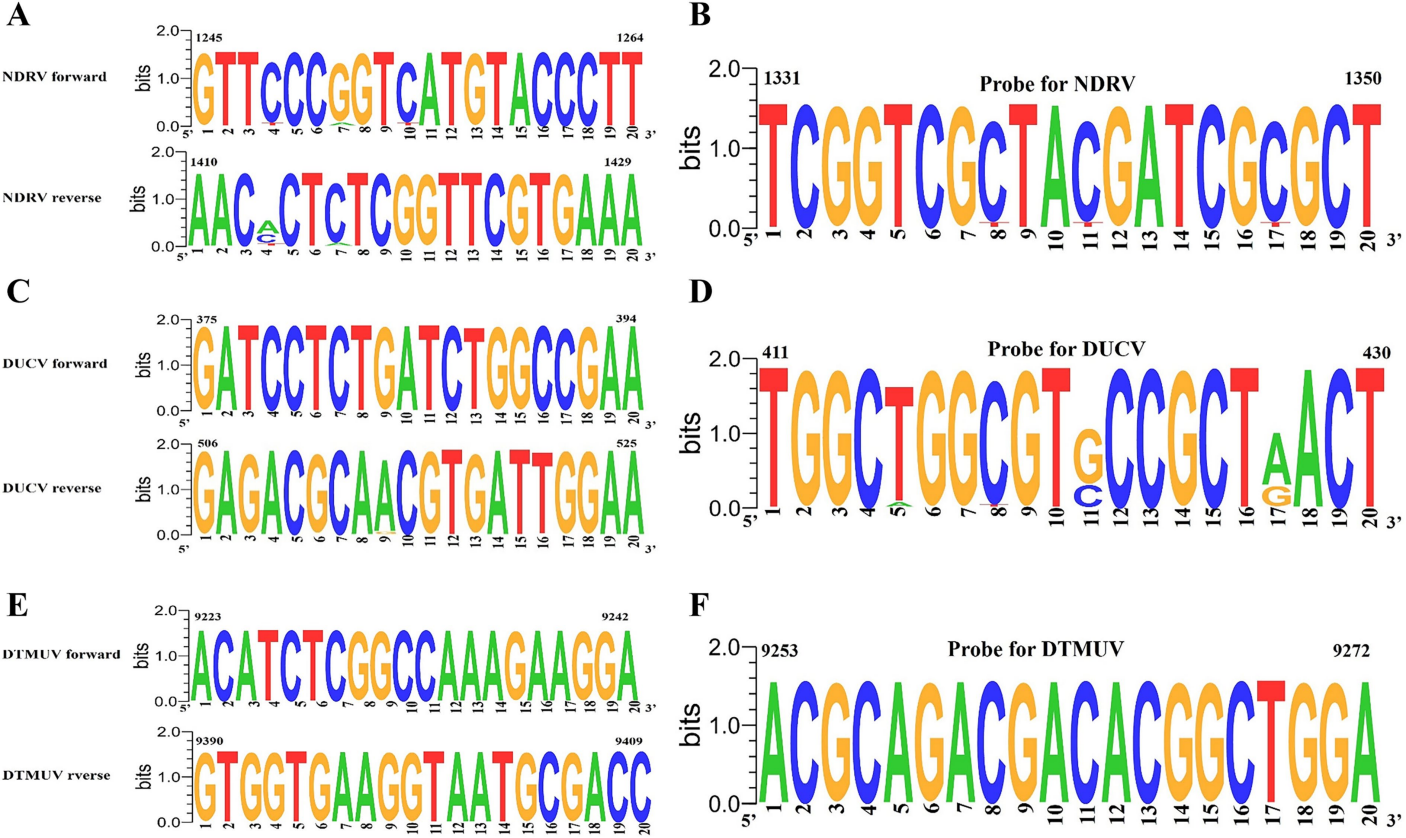
Primers and probes designed in this study. Logo plots show the highly conserved genomic regions of primers and corresponding probes for NDRV **(A**,**B)**, DuCV **(C,D)**, and DTMUV **(E,F)**.

The genomic sequences of DTMUV (*n* = 18), NDRV (*n* = 17), and DuCV (*n* = 94) were retrieved from the GenBank database. The polymerase gene (GenBank: KY623425.1), λA gene (GenBank: MT829224.1), and replication-associated protein gene (GenBank: MN078103.1) were selected as detection targets for DTMUV, NDRV, and DuCV, respectively. Multiple sequence alignments were performed separately for the target genes of each virus using MAFFT software to identify conserved regions within each gene (Figure 1). Specific primers and TaqMan probes were designed within these conserved regions using Oligo 7.0 software. For simultaneous differential detection of the three viruses in a single reaction, the probes were labeled with FAM, VIC, and CY5 for DTMUV, NDRV, and DuCV, respectively, each with a corresponding quencher. All primers and probes were synthesized by Sangon Biotech (Shanghai, China) and stored at −20 °C. Detailed information is provided in Table 1.

**Table 1:** Primers and probes for detection of DTMUV, NDRV, and DuCV.

Name	Sequences (5’ → 3’)	Amplicon size (bp)
DTMUV-F	ACATCTCGGCCAAAGAAGGA	187
DTMUV-R	GGTCGCATTACCTTCACCAC	
DTMUV-probe	FAM-TCCAGCCGTGTCGTCTGCGT-DBQ1	
NDRV-F	GTTCCCGGTCATGTACCCTT	185
NDRV-R	TTTCACGAACCGAGAGGGTT	
NDRV-probe	VIC-TCGGTCGCTACGATCGCGCT-DBQ1	
DuCV-F	GATCCTCTGATCTGGCCGAA	151
DuCV-R	TTCCAATCACGTTGCGTCTC	
DuCV-probe	CY5-TGGCTGGCGTCCCGCTGACT-DBQ1	

### 2.3 Viral nucleic acids extraction and cDNA synthesis

All clinical samples were thoroughly homogenized in phosphate-buffered saline (PBS, pH 7.2), then centrifuged at 4 °C at 12,000 rpm for 5 min, and the supernatant was collected. Viral nucleic acids were extracted using the TaKaRa MiNiBEST Viral RNA/DNA Extraction Kit V5 (TaKaRa, Dalian, China). Viral RNA of DTMUV and NDRV was reverse-transcribed into complementary DNA (cDNA) using the HiScript II Reverse Transcriptase Kit (Vazyme, Nanjing, China). All the above procedures were carried out in accordance with the manufacturer’s instructions. RNA, DNA, and cDNA were stored at −80 °C for subsequent use.

### 2.4 Construction of recombinant plasmids

PCR amplification was performed using the cDNA of DTMUV and NDRV and the DNA of DuCV as templates. The amplified products were recovered and purified, then ligated into the pMD18-T vector and transformed into *E. coli* DH5α competent cells to construct the recombinant plasmid. The concentration of Plasmids was quantified using a Nanodrop spectrophotometer, and the copy number was calculated according to the formula reported by Li et al. (2023).

### 2.5 Optimization of the TaqMan multiplex real-time qPCR assay

The TaqMan multiplex real-time qPCR assay was performed using the ACE QPCR Probe Master Mix (Vazyme, Nanjing, China). Primer and probe concentrations were evaluated using an orthogonal matrix approach to ensure specific amplification at the lowest cycle threshold (Ct) and to obtain high fluorescence intensity signals, thereby determining the optimal concentrations for primers and probes.

### 2.6 Standard curve construction

Prepare single templates with concentrations ranging from 10^8^ to 10^1^ copies/μL by performing 10-fold serial dilutions of the three recombinant plasmids using nuclease-free water. Additionally, prepare a mixed template by combining equal volumes of each dilution level of the single templates. For each dilution level, the single and mixed templates (*n* = 3 per group) were analyzed using the established TaqMan multiplex real-time qPCR assay. The standard curve was generated by plotting the Ct values on the y-axis against the logarithmic values of plasmid copy numbers on the x-axis.

### 2.7 Assessment of assay specificity

The nucleic acid of DTMUV, NDRV and DuCV, and other common viruses, including DPV, NDV, DAdV-4, MDRV, GoAstV-1, GoAstV-2, GPV, AIV, GRV, FAdV, DHAV-1, DHAV-3, DAstV and double-distilled water were tested to evaluate the specificity of the established assay. NDRV, DTMUV and DuCV served as positive controls, whereas ddH₂O served as the negative control.

### 2.8 Assessment of assay sensitivity

To assess the detection sensitivity of this method, the recombinant plasmid of DTMUV, NDRV, and DuCV were mixed at an equal ratio (1:1:1) and then subjected to 10-fold serial dilution. To evaluate potential amplification interference among targets with markedly different template concentrations, three mixed-plasmid combinations were prepared, in which one target plasmid was present at 10^8^ copies/μL and the other two target plasmids were present at 10^1^ copies/μL. Specifically, the combinations were NDRV at 10^8^ copies/μL with DTMUV and DuCV at 10^1^ copies/μL, DTMUV at 10^8^ copies/μL with NDRV and DuCV at 10^1^ copies/μL, and DuCV at 10^8^ copies/μL with DTMUV and NDRV at 10^1^ copies/μL. All combinations were tested using the established multiplex real-time qPCR assay.

### 2.9 Assessment of assay repeatability

To evaluate the repeatability of the TaqMan multiplex real-time qPCR assay, a mixture of the three recombinant plasmids was serially diluted to generate a concentration series ranging from 10^8^ to 10^1^ copies/μL. The mean ( $\bar{x}$ ) and standard deviation (SD) of the test results were calculated for each dilution level. The coefficient of variation (CV) was calculated using the formula: CV (%) = (SD / $\bar{x}$ ) × 100 to evaluate the repeatability of this assay.

### 2.10 Clinical samples collection

To further validate the clinical applicability of the TaqMan multiplex real-time qPCR assay established in this study. A total of 120 liver tissue samples were collected from diseased ducks randomly sampled at five duck farms covering different regions of Jiangxi Province. All samples were tested using the established assay and conventional PCR. Statistical analysis was conducted to single and mixed infection patterns.

## 3 Results

### 3.1 Optimization of the TaqMan multiplex real-time qPCR assay reaction system

Experimental results indicate that the reaction performance of primers and probes varies significantly at different concentrations, with amplification efficiency being highest at 0.2 μM for both primers and probes. The 20 μL multiplex real-time qPCR reaction system was established in this study, including 10 μL 2 × AceQ QPCR Probe Master Mix, 0.4 μL forward and reverse primers, 0.2 μL TaqMan probe, 0.4 μL 50 × ROX Reference Dye 1, adjusted to the final volume of 20 μL with nuclease-free water. The amplification procedure comprises: pre-denaturation at 95 °C for 5 min, followed by 40 cycles, each consisting of 5 s at 95 °C and 30 s at 60 °C. The acquisition of the sample signal was completed at the 60 °C stage.

### 3.2 Standard curve

**Figure 2:**
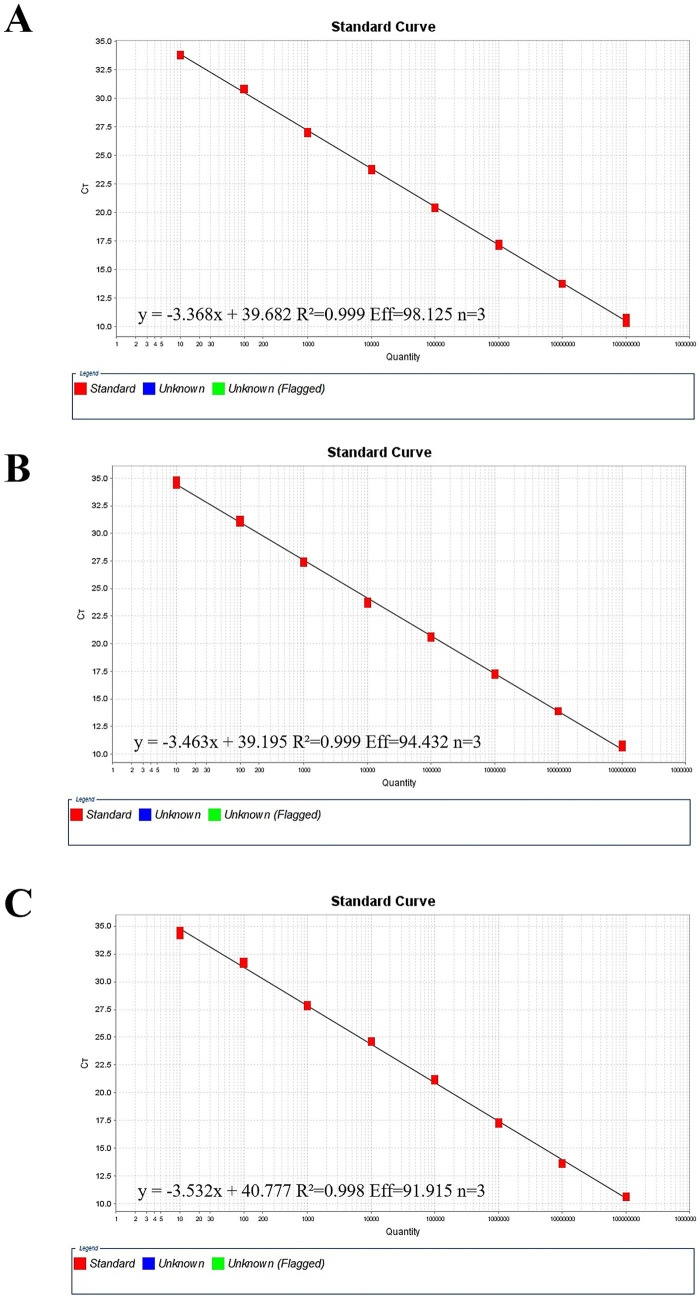
Establishment of standard curve of the individual virus. **(A)** The standard curve of the DTMUV. **(B)** The standard curve of the NDRV. **(C)** The standard curve of the DuCV.

Upon the TaqMan multiplex real-time qPCR assay analysis of the single template, the standard curve demonstrated favorable amplification efficiency and correlation. Specifically, the DTMUV linear regression equation was y = −3.368x + 39.682, with the slope was −3.368, the correlation coefficient (*R*^2^) was 0.999, and the intercept was 39.682 (Figure 2A). The NDRV linear equation exhibits the slope was −3.463, the correlation coefficient (*R*^2^) was 0.999, and the intercept was 39.195, with its linear regression equation being y = −3.463x + 39.195 (Figure 2B). The DuCV linear equation exhibited the slope was −3.532, the correlation coefficient (*R*^2^) was 0.998, and the intercept was 40.777. The standard curve linear regression equation was y = −3.532x + 40.777 (Figure 2C). Subsequently, we performed the TaqMan multiplex real-time qPCR on the mixed template of the three viruses, with all parameters detailed in Table 2. The results demonstrated that the detection levels of the pooled test were comparable to those of individual testing, with no cross-reactivity observed. This indicates that pooled testing does not compromise the sensitivity or specificity of the assay.

**Table 2:** Characteristic parameters of the multiplex real-time qPCR standard curve.

Detection type	Gene	Slope	Y-Inter	*R* ^2^	Eff (%)
Single	DTMUV	−3.368	39.682	0.999	98.125
	NDRV	−3.463	39.195	0.999	94.432
	DuCV	−3.532	40.777	0.998	91.915
Multiplex	DTMUV	−3.341	37.203	1.000	99.194
	NDRV	−3.421	37.818	0.999	96.018
	DuCV	−3.473	38.28	0.999	94.071

### 3.3 Specificity of the TaqMan multiplex real-time qPCR assay

**Figure 3:**
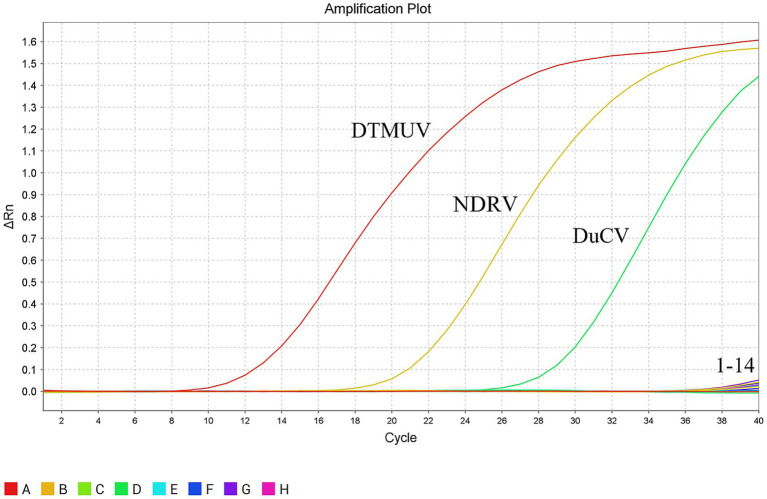
Specificity analysis of the TaqMan multiplex real-time qPCR assay. Numbers 1–14 represent DPV, NDV, DAdV-4, MDRV, GoAstV-1, GoAstV-2, GPV, AIV, GRV, FAdV, DHAV-1, DHAV-3, DAstV and ddH_2_O in turn.

The results of the multiplex real-time qPCR demonstrated specific amplification curves for DTMUV, NDRV, and DuCV, whereas no fluorescent signals were detected for other viruses (Figure 3). This indicates this method exhibits high specificity for these three viruses.

### 3.4 Sensitivity of the TaqMan multiplex real-time qPCR assay

**Figure 4:**
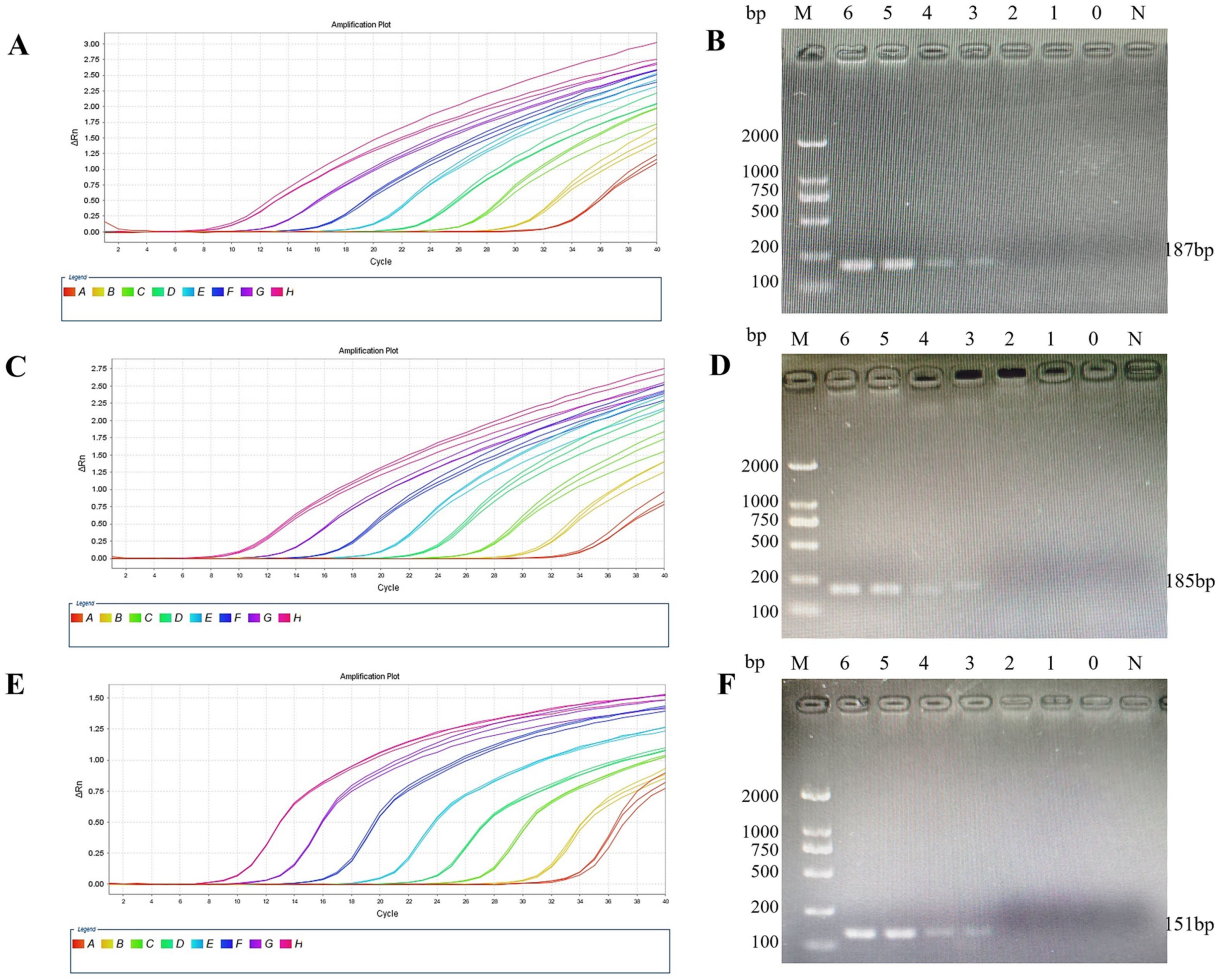
Sensitivity analysis of the TaqMan multiplex real-time qPCR assay. **(A,B)**: DTMUV; **(C,D)**: NDRV; **(E,F)**: DuCV. A to H means standard template at 10^1^–10^8^ copies/μL in turn. 6 ~ 0 means the concentration of 10^6^–10^0^ copies/μL in turn; M means marker; N means negative control.

To assess the sensitivity of the multiplex real-time qPCR assay, three recombinant plasmids were serially diluted in a 10-fold series (10^8^ to 10^1^ copies/μL) and employed as detection templates. Results indicate that the limit of detection (LOD) of the conventional PCR assay were determined to be 10^3^ copies/μL for DTMUV, NDRV, and DuCV. By comparison, the multiplex real-time qPCR assay exhibits the detection limit of 10^1^ copies/μL for all three viruses, representing a 100-fold increase in sensitivity compared with conventional PCR (Figure 4). The above results confirm that this method possesses high sensitivity. In addition, testing using mixed plasmid templates showed that the presence of one target plasmid at a high copy number (10^8^ copies/μL) did not compromise the detection of the other two target plasmids at low copy numbers (10^1^ copies/μL) under the tested conditions (Supplementary Figure S1).

### 3.5 Repeatability of the TaqMan multiplex real-time qPCR assay

As shown in Table 3, the CV for DTMUV, NDRV, and DuCV ranged from 0.23 to 0.53, 0.31 to 1.08, and 0.29 to 1.53 respectively, with all CV values falling within 2%. The results indicate that the multiplex real-time qPCR assay established in this study possesses good reproducibility and reliability.

**Table 3:** Repeatability test of the multiplex real-time qPCR.

Name	Number of standard templates (copies/μL)	Mean	SD	CV (%)
DTMUV	10^1^	34.96	0.14	0.39
	10^2^	32.50	0.10	0.31
	10^3^	29.43	0.08	0.27
	10^4^	26.25	0.06	0.23
	10^5^	22.82	0.09	0.41
	10^6^	19.60	0.08	0.41
	10^7^	16.21	0.06	0.39
	10^8^	12.43	0.07	0.53
NDRV	10^1^	34.95	0.36	1.02
	10^2^	31.50	0.10	0.31
	10^3^	27.46	0.12	0.45
	10^4^	24.93	0.13	0.51
	10^5^	21.76	0.08	0.36
	10^6^	18.41	0.09	0.47
	10^7^	15.27	0.14	0.93
	10^8^	11.52	0.12	1.08
DuCV	10^1^	35.2	0.26	0.72
	10^2^	32.57	0.26	0.71
	10^3^	29.48	0.15	0.53
	10^4^	27.13	0.25	0.93
	10^5^	23.45	0.13	0.57
	10^6^	19.55	0.08	0.39
	10^7^	15.86	0.05	0.29
	10^8^	12.30	0.19	1.53

### 3.6 Clinical sample detection

As shown in Table 4, single and mixed infections of NDRV, DTMUV, and DuCV were detected among the 120 samples. For single infections, the detection rate of NDRV was the highest at 30.0%, followed by DTMUV and DuCV with rates of 18.3 and 6.7%, respectively. Among mixed infections, co-infection with NDRV and DTMUV, NDRV and DuCV, and DTMUV and DuCV were 6.7, 3.3, and 1.7%, respectively. Additionally, triple infection of all three viruses was also observed, with a detection rate of 0.83%. In comparison, conventional PCR exhibits a notably lower detection rate for the three viruses and fails to identify most mixed infections. Accordingly, this method features higher sensitivity and superior capability to distinguish mixed infections accurately, making it suitable for the simultaneous detection of the three pathogens.

**Table 4:** Clinical sample detection by multiplex qPCR assay and comparison with conventional PCR.

Virus	Number of clinical samples	qPCR	Conventional PCR
NDRV	120	30.0% (36/120)	18.3% (22/120)
DTMUV	120	18.3% (22/120)	12.5% (15/120)
DuCV	120	6.7% (8/120)	5% (6/120)
NDRV+DTMUV	120	6.7% (8/120)	1.7% (2/120)
NDRV+DuCV	120	3.3% (4/120)	0.83% (1/120)
DTMUV+DuCV	120	1.7% (2/120)	0.83% (1/120)
NDRV+DTMUV+DuCV	120	0.83% (1/120)	0 (0/120)

## 4 Discussion

DTMUV, NDRV, DuCV are currently common pathogens severely threatening the healthy development of China’s waterfowl farming industry (Cheng et al., 2024; Liu et al., 2020; Yan et al., 2021). They often present as mixed infections, with overlapping clinical symptoms and pathological changes, making it difficult to distinguish them based on gross examination (Yin et al., 2023). Currently, multiple detection technologies have been established for NDRV, DTMUV, and DuCV, including virus isolation and identification (Kong et al., 2023), conventional PCR (Fringuelli et al., 2006), Enzyme-Linked Immunosorbent Assay (ELISA) (Liu et al., 2010; Putchong et al., 2025), and real-time quantitative PCR (Da et al., 2021; Zhang et al., 2020). These methods provide important technical support for the detection and identification of the three pathogens. Nevertheless, these methods are predominantly applied for single-virus detection. In contrast, the multiplex real-time qPCR assay overcomes this limitation. It enables rapid, accurate and sensitive detection of multiple pathogens in a single reaction system, which is of great significance for the rapid diagnosis, prevention and epidemiological research of mixed infections.

This study designed primers and probes based on the highly conserved regions of the NDRV, DTMUV, and DuCV viruses. Through systematic optimization of primer and probe concentrations, a TaqMan multiplex real-time qPCR assay was successfully established. Notably, this method employs three distinct fluorescent reporter groups (FAM, VIC, CY5) to achieve efficient differentiation and precise identification of these three pathogens within a single reaction. The method established in this study achieved the limit of detection of 10^1^ copies/μL for all three viruses, which is consistent with previously reported duck multiplex real-time qPCR assays (Gan et al., 2026; Wang et al., 2020; Zhang et al., 2020), while demonstrating a 100-fold increase in sensitivity compared to conventional PCR. This effectively addresses the shortcomings of conventional PCR, which is prone to false negatives. It should be noted that, although multiplex digital PCR (dPCR) achieves 10-fold higher sensitivity than qPCR, multiplex reaction conditions limit its clinical application (Yin et al., 2023). On the one hand, the small reaction volume of dPCR restricts its utility for high-throughput pathogen screening of clinical samples. On the other hand, this technique requires expensive instruments and reagents, involves complicated operational procedures, and carries an elevated risk of cross-contamination between samples.

Repeatability testing is a critical step in ensuring the accuracy and reliability of experimental results (Qiu et al., 2025). During the development of a multiplex RT-qPCR method, the presence of multiple primer-probe sets often leads to signal crosstalk between different reactions, while sample cross-contamination may also occur within a single reaction (Taylor et al., 2019). In the present study, the developed method achieved a CV of below 1% for both NDRV and DTMUV across the concentration range of 10^4^–10^7^ copies/μL, demonstrating excellent reproducibility. Finally, the multiplex detection method established in this study was used to screen 120 clinical samples collected in Jiangxi Province. The positive detection rates of NDRV, DTMUV and DuCV were 30.0, 18.3 and 6.7%, respectively, and co-infection cases of the three viruses were identified. In contrast, conventional PCR yielded much lower detection rates of 18.3, 12.5 and 5.0% for the three pathogens, and exhibited poor capability to detect co-infected samples. Liver tissue was selected as the test specimen based on the tissue tropism of the three pathogens (Kong et al., 2023; Lei et al., 2024; Yin et al., 2025). From the perspective of clinical pathological features, no typical lesions were observed in samples infected with DuCV and DTMUV in this study, while obvious necrosis of the liver and spleen was found in samples infected with NDRV. Notably, previous studies have shown that the clinical manifestations of NDRV exhibit obvious host dependence (Zeng et al., 2025): it causes typical lesions such as severe necrosis of the liver and spleen in mule ducks, whereas it mostly presents as asymptomatic infection in shelducks. This finding indicates that it is hard to differentially diagnose the above diseases merely based on clinical symptoms and necropsy lesions. In addition, infection with immunosuppressive pathogens can suppress host immune function, which greatly predisposes animals to secondary and mixed infections. Therefore, the detection method established in this study can provide important technical support for the rapid identification the three viruses and scientific prevention and control of waterfowl pathogens.

In summary, this study established a TaqMan-based multiplex real-time qPCR assay for the simultaneous detection of DTMUV, NDRV, and DuCV. The assay showed good specificity, sensitivity, and repeatability. It was able to detect single and mixed infections in clinical samples more effectively than conventional PCR. These results indicate that the method may serve as a useful laboratory tool for the rapid diagnosis of these three duck viruses, especially for suspected co-infections. However, the clinical evaluation in this study was based on a limited number of samples from Jiangxi Province. Further testing with larger field sample sets from different regions, duck ages, and farming conditions will be needed to better assess the practical value and applicability of this assay.

## Data Availability

The original contributions presented in the study are included in the article/Supplementary material, further inquiries can be directed to the corresponding author/s.

## Supplementary material

Supplementary Figure S1 Interference analysis of high-titer targets on low-titer targets in the real-time multiplex qPCR system. **(A)** Mixed plasmid templates containing NDRV at 10⁸ copies/μL, DTMUV and DuCV each at 10¹ copies/μL; **(B)** Mixed plasmid templates containing DTMUV at 10⁸ copies/μL, NDRV and DuCV each at 10¹ copies/μL; **(C)** Mixed plasmid templates containing DuCV at 10⁸ copies/μL, DTMUV and NDRV each at 10¹ copies/μL.

Supplementary Figure S2 Cross-reactivity evaluation of the real-time multiplex qPCR assay. **(A)**: DTMUV; **(B)**: NDRV; **(C)**: DuCV.; A to H means standard template at 101-108 copies/μL in turn.
