# Supplementary material for: TaqMan-probe-based multiplex real-time qPCR assay for simultaneous detection of Duck Tembusu virus, Novel duck reovirus, and Duck circovirus
Source: Front Microbiol. 2026 Jul 21;17:1884322. doi: 10.3389/fmicb.2026.1884322 (PMC13433525; doi:10.3389/fmicb.2026.1884322)
Supplement: Supplementary file 1 [file Table_1.DOCX]

**Table S1 Reference NDRV strains used in this study**

| **No.** | **Strain** | **Genbank** | **Genotype** | **Time** | **Location** | **Host** |
| --- | --- | --- | --- | --- | --- | --- |
| 1 | SD19 | MT829224.1 | NDRV | 2019 | China | Duck |
| 2 | DE150 | MT939501.1 | DRV | 2020 | China | Duck |
| 3 | 091 | JX478250.1 | DRV | 2009 | China | Duck |
| 4 | HN5d | KT861587.1 | DRV | 2013 | China | Duck |
| 5 | SH12 | MH510245.1 | MDRV | 2012 | China | Duck |
| 6 | DH13 | MH510255.1 | MDRV | 2013 | China | Duck |
| 7 | GX-Y7 | MN747004.1 | DRV | 2018 | China | Duck |
| 8 | XT18 | MK749398.1 | ARV | 2018 | China | Duck |
| 9 | SY | MK955818.1 | DRV | 2018 | China | Duck |
| 10 | LY20 | ON040907.1 | ARV | 2021 | China | Duck |
| 11 | QR | MZ736881.1 | DRV | 2020 | China | Duck |
| 12 | YL | MZ733729.1 | DRV | 2020 | China | Duck |
| 13 | TH11 | KC493572.1 | DRV | 2011 | China | Anas platyrhynchos |
| 14 | SD19 | MT829214.1 | DRV | 2019 | China | Cherry Valley Duck |
| 15 | J18 | JX478260.2 | MDRV | 2008 | China | Muscovy duck |
| 16 | ZJ00M | KF154110.1 | DRV | 2000 | China | Duck |
| 17 | SD-12 | KJ879924.1 | DRV | 2012 | China | mallard wild duck |
