# Supplementary material for: TaqMan-probe-based multiplex real-time qPCR assay for simultaneous detection of Duck Tembusu virus, Novel duck reovirus, and Duck circovirus
Source: Front Microbiol. 2026 Jul 21;17:1884322. doi: 10.3389/fmicb.2026.1884322 (PMC13433525; doi:10.3389/fmicb.2026.1884322)
Supplement: Supplementary file 2 [file Table_2.DOCX]

**Table S2 Reference DuCV strains used in this study**

| **No.** | **Strain** | **Genbank** | **Genotype** | **Time** | **Location** | **Host** |
| --- | --- | --- | --- | --- | --- | --- |
| 1 | SD17 | MN078101.1 | DuCV-1 | 2017 | China | Duck |
| 2 | SDLY0201 | MF627690.1 | DuCV-1 | 2016 | China | Duck |
| 3 | SDDY0520 | MF627689.1 | DuCV-1 | 2016 | China | Cherry Valley duck |
| 4 | HN05 | MN928799.1 | DuCV | 2019 | China | Duck |
| 5 | ZJ02 | MN928793.1 | DuCV | 2018 | China | Duck |
| 6 | MH25 | EF451157.1 | DuCV | 2007 | China | Duck |
| 7 | WF0801 | GU131340.1 | DuCV | 2008 | China | Cherry valley duck |
| 8 | DU91 | HM162345.1 | DuCV | 2009 | China | Cherry valley duck |
| 9 | FJZZ302 | GQ423747.1 | DuCV | 2008 | China | Duck |
| 10 | FJMH207 | GQ423742.1 | DuCV | 2007 | China | Duck |
| 11 | FC32 | KC460524.1 | DuCV | 2012 | China | Duck |
| 12 | NN13 | KC460532.1 | DuCV | 2012 | China | Duck |
| 13 | GX190512 | MK814583.1 | DuCV | 2019 | China | Muscovy duck |
| 14 | FujianZQ300 | GQ423740.1 | DuCV | 2008 | China | Muscovy duck |
| 15 | FJzq290 | GU168779.1 | DuCV | 2008 | China | Muscovy duck |
| 16 | LZ04 | KC460529.1 | DuCV | 2011 | China | Duck |
| 17 | ZZ170302 | MN068355.1 | DuCV | 2017 | China | Cherry Valley Duck |
| 18 | LZ/11/09 | HQ180265.1 | DuCV | 2009 | China | Muscovy duck |
| 19 | LS01 | KP780079.1 | DuCV | 2014 | China | Muscovy duck |
| 20 | ShijiazhuangNO.3 | MW255979.1 | DuCV | 2019 | China | Duck |
| 21 | HN06 | MN928800.1 | DuCV | 2019 | China | Duck |
| 22 | LA-18 | MT799793.1 | DuCV | 2018 | China | Duck |
| 23 | DU102 | HM162351.1 | DuCV | 2010 | China | Cherry valley duck |
| 24 | DU104 | HM162353.1 | DuCV | 2010 | China | Cherry valley duck |
| 25 | DB46-12 | KP229369.1 | DuCV | 2013 | China | pekin duck |
| 26 | DB41-18 | KP229368.1 | DuCV | 2013 | China | pekin duck |
| 27 | D12-KD-028 | KC851821.1 | DuCV | 2012 | South Korea | Duck |
| 28 | D12-JW-059 | KC851816.1 | DuCV | 2012 | South Korea | Duck |
| 29 | D12-JW-035 | KC851814.1 | DuCV | 2012 | South Korea | Duck |
| 30 | D11-JW-013 | KC851808.1 | DuCV | 2011 | South Korea | Duck |
| 31 | D11-JW-022 | KC851810.1 | DuCV | 2011 | South Korea | Duck |
| 32 | D11-JW-006 | JQ740362.1 | DuCV | 2011 | South Korea | Duck |
| 33 | D12-JW-047 | KC851815.1 | DuCV | 2012 | South Korea | Duck |
| 34 | D11-JW-024 | KC851811.1 | DuCV | 2011 | South Korea | Duck |
| 35 | D11-JW-015 | KC851809.1 | DuCV | 2011 | South Korea | Duck |
| 36 | D12-KD-019 | KC851819.1 | DuCV | 2012 | South Korea | Duck |
| 37 | D11-JW-009 | KC851806.1 | DuCV | 2011 | South Korea | Duck |
| 38 | D11-JW-007 | KC851805.1 | DuCV | 2011 | South Korea | Duck |
| 39 | D11-JW-010 | KC851807.1 | DuCV | 2011 | South Korea | Duck |
| 40 | D12-KD-027 | KC851820.1 | DuCV | 2012 | South Korea | Duck |
| 41 | 01-Sep-2011 | KC851813.1 | DuCV | 2011 | South Korea | Duck |
| 42 | 01-Sep-2012 | KC851822.1 | DuCV | 2012 | South Korea | Duck |
| 42 | D12-KD-002 | KC851818.1 | DuCV | 2012 | South Korea | Duck |
| 43 | D11-JW-001 | JQ740360.1 | DuCV | 2011 | South Korea | Duck |
| 44 | D11-JW-004 | KC851804.1 | DuCV | 2011 | South Korea | Duck |
| 45 | D11-JW-002 | JQ740361.1 | DuCV | 2011 | South Korea | Duck |
| 46 | SDHZ1223 | MT084134.1 | DuCV | 2018 | China | Anas platyrhynchos |
| 47 | YF180403 | MN068360.1 | DuCV | 2017 | China | Cherry Valley Duck |
| 48 | HZ170301 | MN068356.1 | DuCV | 2017 | China | Cherry Valley Duck |
| 49 | GD/ZQ/144 | ON227548.1 | DuCV | 2021 | China | Duck |
| 50 | YF180401 | MN068357.1 | DuCV | 2017 | China | Cherry Valley  Duck |
| 51 | GD/QY/145 | ON227549.1 | DuCV | 2021 | China | Duck |
| 52 | BaodingNO.Du | MW255980.1 | DuCV | 2019 | China | Duck |
| 53 | GD/QY/129 | ON227542.1 | DuCV | 2021 | China | Duck |
| 54 | GD/QY/151 | ON227550.1 | DuCV | 2021 | China | Duck |
| 55 | GD/FS/132 | ON227544.1 | DuCV | 2020 | China | Duck |
| 56 | ZZ170301 | MN068354.1 | DuCV | 2017 | China | Cherry Valley Duck |
| 57 | GD/JM/180 | ON227551.1 | DuCV | 2021 | China | Duck |
| 58 | GD/ZQ/188 | ON227553.1 | DuCV | 2021 | China | Duck |
| 59 | GD/FS/125 | ON227538.1 | DuCV | 2021 | China | Duck |
| 60 | GD/FS/184 | ON227552.1 | DuCV | 2021 | China | Duck |
| 61 | GD/ZQ/143 | ON227547.1 | DuCV | 2021 | China | Duck |
| 62 | GD/FS/128 | ON227541.1 | DuCV | 2021 | China | Duck |
| 63 | HN01 | MN928794.1 | DuCV | 2018 | China | Duck |
| 64 | GD/JM/244 | ON227554.1 | DuCV | 2021 | China | Duck |
| 65 | AHAUHQ | MT646350.1 | DuCV | 2019 | China | Duck |
| 66 | GD/JM/142 | ON227546.1 | DuCV | 2021 | China | Duck |
| 67 | HN04 | MN928798.1 | DuCV | 2019 | China | Duck |
| 68 | ZJ03 | MN928797.1 | DuCV | 2018 | China | Duck |
| 69 | WF190501 | MN068359.1 | DuCV | 2017 | China | Cherry Valley Duck |
| 70 | HN07 | MN928801.1 | DuCV | 2019 | China | Duck |
| 71 | GD/JM/131 | ON227543.1 | DuCV | 2021 | China | Duck |
| 72 | WF0803 | GU131342.1 | DuCV | 2008 | China | Cherry Valley Duck |
| 73 | WF0701 | EU022375.1 | DuCV | 2021 | China | Duck |
| 74 | NN11/2012 | KC460530.1 | DuCV | 2012 | China | Duck |
| 75 | GX180512 | MK814579.1 | DuCV | 2018 | China | Mscovy duck |
| 76 | DB32-3 | KP229367.1 | DuCV | 2013 | China | Pekin duck |
| 77 | HN02 | MN928795.1 | DuCV | 2018 | China | Duck |
| 78 | GD/ZQ/141 | ON227545.1 | DuCV | 2021 | China | Duck |
| 79 | JSPX03E | MF627688.1 | DuCV-1 | 2016 | China | Cherry Valley duck |
| 80 | AH01 | MN928808.1 | DuCV | 2018 | China | Duck |
| 81 | AH02 | MN928809.1 | DuCV | 2018 | China | Duck |
| 82 | SDFC12 | KY328304.1 | DuCV | 2015 | China | Cherry valley duck |
| 83 | PX08/2011 | KC460533.1 | DuCV | 2011 | China | Duck |
| 84 | D11-JW-008 | JQ740363.1 | DuCV | 2011 | South Korea | Duck |
| 85 | D12-KD-001 | KC851817.1 | DuCV | 2012 | South Korea | Duck |
| 86 | GD/JM/127 | ON227540.1 | DuCV | 2021 | China | Duck |
| 87 | YF180404 | MN068358.1 | DuCV | 2017 | China | Cherry Valley duck |
| 88 | GD/ZQ/99 | ON227537.1 | DuCV | 2021 | China | Duck |
| 89 | FC33/2012 | KC460525.1 | DuCV | 2012 | China | Duck |
| 90 | FC34/2012 | KC460526.1 | DuCV | 2012 | China | Duck |
| 91 | JSPX03B | MF627687.1 | DuCV-1 | 2016 | China | Cherry Valley duck |
| 92 | HB18 | MN078103.1 | DuCV-1 | 2018 | China | Duck |
| 93 | TD/41/09 | HQ180266.1 | DuCV | 2009 | China | Mulard duck |
| 94 | JSXZ0209 | MT084133.1 | DuCV | 2018 | China | Anas platyrhynchos |
