# Supplementary material for: TaqMan-probe-based multiplex real-time qPCR assay for simultaneous detection of Duck Tembusu virus, Novel duck reovirus, and Duck circovirus
Source: Front Microbiol. 2026 Jul 21;17:1884322. doi: 10.3389/fmicb.2026.1884322 (PMC13433525; doi:10.3389/fmicb.2026.1884322)
Supplement: Supplementary file 3 [file Table_3.DOCX]

**Table S3 Reference DTMUV strains used in this study**

| **No.** | **Strain** | **Genbank** | **Genotype** | **Time** | **Location** | **Host** |
| --- | --- | --- | --- | --- | --- | --- |
| 1 | SH201002 | KY623442.1 | DTMUV | 2010 | China | Duck |
| 2 | NMCF | MH764607.1 | DTMUV | 2017 | China | Meat ducks |
| 3 | xz 2012 | KM188953.1 | DTMUV | 2012 | China | Duck |
| 4 | P4-5b | OK315317.1 | DTMUV | 2021 | China | Duck |
| 5 | lq-1 | KF557893.1 | TMUV | 2012 | China | Wulong goose |
| 6 | WU2016 | OQ920272.1 | TMUV | 2016 | China | Duck |
| 7 | WJ-1 | JX549382.1 | TMUV | 2012 | China | Muscovy duck |
| 8 | LD_2010 | KC990544.1 | DTMUV | 2010 | China | Duck |
| 9 | FS-2011 | KX686578.1 | TMUV | 2011 | China | layer duck |
| 10 | SX1 | KM066945.1 | TMUV | 2013 | China | Layer chicken |
| 11 | P4-3b | OK315314.1 | TMUV | 2021 | China |  |
| 12 | PS | KT876991.1 | TMUV | 2015 | China | Duck |
| 13 | 2021HN | OR215429.1 | TMUV | 2021 | China | Chicken |
| 14 | FS | JN811558.1 | TMUV | 2011 | China | Duck |
| 15 | FX2010 | MH414568.1 | TMUV | 2010 | China | shelduck |
| 16 | SDDZ | MH764606.1 | TMUV | 2016 | China | meat ducks |
| 17 | TMUV-JS06 | KR869106.1 | TMUV | 2014 | China | Chicken |
| 18 | YY5 | JF270480.1 | TMUV | 2010 | China | Duck |
