# Supplementary material for: TaqMan-probe-based multiplex real-time qPCR assay for simultaneous detection of Duck Tembusu virus, Novel duck reovirus, and Duck circovirus
Source: Front Microbiol. 2026 Jul 21;17:1884322. doi: 10.3389/fmicb.2026.1884322 (PMC13433525; doi:10.3389/fmicb.2026.1884322)
Supplement: Supplementary file 4 [file Table_4.DOCX]

**Table S4 Ct values of different waterfowl pathogens in specificity assay**

| **No.** | **Pathogen species** | **CT** |
| --- | --- | --- |
| 1 | NDRV | 13.05 |
| 2 | DTMUV | 21.40 |
| 3 | DuCV | 29.18 |
| 4 | DPV | Undetermined |
| 5 | NDV | Undetermined |
| 6 | DAdV-4 | Undetermined |
| 7 | MDRV | Undetermined |
| 8 | GoAstV-1 | Undetermined |
| 9 | GoAstV-2 | Undetermined |
| 10 | GPV | Undetermined |
| 11 | AIV | Undetermined |
| 12 | GRV | Undetermined |
| 13 | FAdV | 39.36 |
| 14 | ddH_2_O | Undetermined |
| 15 | DHAV-1 | Undetermined |
| 16 | DHAV-3 | Undetermined |
| 17 | DAstV | Undetermined |
