# Supplementary material for: TaqMan-probe-based multiplex real-time qPCR assay for simultaneous detection of Duck Tembusu virus, Novel duck reovirus, and Duck circovirus
Source: Front Microbiol. 2026 Jul 21;17:1884322. doi: 10.3389/fmicb.2026.1884322 (PMC13433525; doi:10.3389/fmicb.2026.1884322)
Supplement: Supplementary file 5 [file Table_5.DOCX]

**Table S5 Ct values of serially diluted DTMUV, NDRV and DuCV recombinant plasmids for sensitivity assay**

| **Plasmid** | **Plasmid dilution** | **CT** |
| --- | --- | --- |
| DTMUV | 10^8^ | 8.84 |
|  |  | 8.83 |
|  |  | 8.71 |
|  | 10^7^ | 12.53 |
|  |  | 12.46 |
|  |  | 12.30 |
|  | 10^6^ | 15.60 |
|  |  | 16.78 |
|  |  | 15.66 |
|  | 10^5^ | 19.89 |
|  |  | 20.70 |
|  |  | 19.86 |
|  | 10^4^ | 24.44 |
|  |  | 24.27 |
|  |  | 25.45 |
|  | 10^3^ | 26.98 |
|  |  | 26.57 |
|  |  | 27.50 |
|  | 10^2^ | 29.89 |
|  |  | 30.30 |
|  |  | 30.02 |
|  | 10^1^ | 31.82 |
|  |  | 32.58 |
|  |  | 32.64 |
| NDRV | 10^8^ | 9.01 |
|  |  | 8.82 |
|  |  | 8.95 |
|  | 10^7^ | 12.75 |
|  |  | 12.58 |
|  |  | 12.50 |
|  | 10^6^ | 15.65 |
|  |  | 15.63 |
|  |  | 15.54 |
|  | 10^5^ | 19.14 |
|  |  | 18.90 |
|  |  | 18.98 |
|  | 10^4^ | 22.32 |
|  |  | 22.23 |
|  |  | 21.85 |
|  | 10^3^ | 25.72 |
|  |  | 25.68 |
|  |  | 25.54 |
|  | 10^2^ | 29.56 |
|  |  | 29.81 |
|  |  | 29.46 |
|  | 10^1^ | 32.74 |
|  |  | 33.17 |
|  |  | 33.71 |
| DuCV | 10^8^ | 9.24 |
|  |  | 9.35 |
|  |  | 9.64 |
|  | 10^7^ | 12.55 |
|  |  | 12.63 |
|  |  | 12.60 |
|  | 10^6^ | 16.32 |
|  |  | 16..21 |
|  |  | 16.52 |
|  | 10^5^ | 20.20 |
|  |  | 19.84 |
|  |  | 19.79 |
|  | 10^4^ | 23.37 |
|  |  | 23.73 |
|  |  | 23.37 |
|  | 10^3^ | 26.55 |
|  |  | 26.71 |
|  |  | 26.23 |
|  | 10^2^ | 30.49 |
|  |  | 30.43 |
|  |  | 30.56 |
|  | 10^1^ | 33.41 |
|  |  | 33.24 |
|  |  | 32.84 |
