# Supplementary material for: TaqMan-probe-based multiplex real-time qPCR assay for simultaneous detection of Duck Tembusu virus, Novel duck reovirus, and Duck circovirus
Source: Front Microbiol. 2026 Jul 21;17:1884322. doi: 10.3389/fmicb.2026.1884322 (PMC13433525; doi:10.3389/fmicb.2026.1884322)
Supplement: Supplementary file 6 [file Table_6.DOCX]

**Table S6 Ct values of serially diluted DTMUV, NDRV and DuCV recombinant plasmids for repeatability assay**

| **Name** | **Number of standard templates**  **(copies/μL)** | **Mean** | **SD** | **CV (%)** |
| --- | --- | --- | --- | --- |
| DTMUV | 10^1^ | 34.83 | 0.14 | 0.39 |
|  |  | 34.95 |  |  |
|  |  | 35.10 |  |  |
|  | 10^2^ | 32.52 | 0.10 | 0.31 |
|  |  | 32.59 |  |  |
|  |  | 32.39 |  |  |
|  | 10^3^ | 29.35 | 0.08 | 0.27 |
|  |  | 29.44 |  |  |
|  |  | 29.50 |  |  |
|  | 10^4^ | 26.23 | 0.06 | 0.23 |
|  |  | 26.20 |  |  |
|  |  | 26.32 |  |  |
|  | 10^5^ | 22.85 | 0.09 | 0.39 |
|  |  | 22.72 |  |  |
|  |  | 22.89 |  |  |
|  | 10^6^ | 19.51 | 0.08 | 0.41 |
|  |  | 19.62 |  |  |
|  |  | 19.67 |  |  |
|  | 10^7^ | 16.23 | 0.06 | 0.37 |
|  |  | 16.14 |  |  |
|  |  | 16.26 |  |  |
|  | 10^8^ | 12.37 | 0.07 | 0.53 |
|  |  | 12.42 |  |  |
|  |  | 12.50 |  |  |
| NDRV | 10^1^ | 34.82 | 0.36 | 1.02 |
|  |  | 34.90 |  |  |
|  |  | 35.13 |  |  |
|  | 10^2^ | 31.39 | 0.10 | 0.31 |
|  |  | 31.54 |  |  |
|  |  | 31.57 |  |  |
|  | 10^3^ | 27.33 | 0.12 | 0.45 |
|  |  | 27.48 |  |  |
|  |  | 27.57 |  |  |
|  | 10^4^ | 24.79 | 0.13 | 0.51 |
|  |  | 24.93 |  |  |
|  |  | 25.05 |  |  |
|  | 10^5^ | 21.66 | 0.08 | 0.36 |
|  |  | 21.82 |  |  |
|  |  | 21.79 |  |  |
|  | 10^6^ | 18.32 | 0.09 | 0.47 |
|  |  | 18.43 |  |  |
|  |  | 18.49 |  |  |
|  | 10^7^ | 15.12 | 0.14 | 0.93 |
|  |  | 15.29 |  |  |
|  |  | 15.40 |  |  |
|  | 10^8^ | 11.54 | 0.12 | 1.08 |
|  |  | 11.39 |  |  |
|  |  | 11.63 |  |  |
| DuCV | 10^1^ | 34.94 | 0.26 | 0.72 |
|  |  | 35.21 |  |  |
|  |  | 35.45 |  |  |
|  | 10^2^ | 32.30 | 0.26 | 0.71 |
|  |  | 32.59 |  |  |
|  |  | 32.82 |  |  |
|  | 10^3^ | 29.31 | 0.15 | 0.53 |
|  |  | 29.49 |  |  |
|  |  | 29.63 |  |  |
|  | 10^4^ | 26.85 | 0.25 | 0.93 |
|  |  | 27.20 |  |  |
|  |  | 27.33 |  |  |
|  | 10^5^ | 23.30 | 0.13 | 0.57 |
|  |  | 23.48 |  |  |
|  |  | 23.56 |  |  |
|  | 10^6^ | 19.47 | 0.08 | 0.39 |
|  |  | 19.55 |  |  |
|  |  | 19.63 |  |  |
|  | 10^7^ | 15.81 | 0.05 | 0.29 |
|  |  | 15.86 |  |  |
|  |  | 15.90 |  |  |
|  | 10^8^ | 12.11 | 0.19 | 1.53 |
|  |  | 12.30 |  |  |
|  |  | 12.48 |  |  |
