# Supplementary material for: TaqMan-probe-based multiplex real-time qPCR assay for simultaneous detection of Duck Tembusu virus, Novel duck reovirus, and Duck circovirus
Source: Front Microbiol. 2026 Jul 21;17:1884322. doi: 10.3389/fmicb.2026.1884322 (PMC13433525; doi:10.3389/fmicb.2026.1884322)
Supplement: Supplementary file 7 [file Table_7.DOCX]

**Table S7 Ct values detected in clinical positive samples**

| No. | NDRV | DTMUV | DuCV |
| --- | --- | --- | --- |
| 1 | 23.68 | Undetermined | Undetermined |
| 2 | 27.65 | Undetermined | Undetermined |
| 3 | 25.36 | Undetermined | Undetermined |
| 4 | 30.24 | Undetermined | Undetermined |
| 5 | 25.65 | Undetermined | Undetermined |
| 6 | 31.23 | Undetermined | Undetermined |
| 7 | 32.24 | Undetermined | Undetermined |
| 8 | 22.68 | Undetermined | Undetermined |
| 9 | 30.12 | Undetermined | Undetermined |
| 10 | 25.63 | Undetermined | Undetermined |
| 11 | 26.65 | Undetermined | Undetermined |
| 12 | 23.89 | Undetermined | Undetermined |
| 13 | 23.29 | Undetermined | Undetermined |
| 14 | 30.82 | Undetermined | Undetermined |
| 15 | 32.26 | Undetermined | Undetermined |
| 16 | 28.26 | Undetermined | Undetermined |
| 17 | 27.58 | Undetermined | Undetermined |
| 18 | 29.63 | Undetermined | Undetermined |
| 19 | 28.54 | Undetermined | Undetermined |
| 20 | 24.52 | Undetermined | Undetermined |
| 21 | 26.68 | Undetermined | Undetermined |
| 22 | 30.36 | Undetermined | Undetermined |
| 23 | 34.35 | Undetermined | Undetermined |
| 24 | Undetermined | 25.36 | Undetermined |
| 25 | Undetermined | 30.96 | Undetermined |
| 26 | Undetermined | 32.45 | Undetermined |
| 27 | Undetermined | 33.68 | Undetermined |
| 28 | Undetermined | 26.76 | Undetermined |
| 29 | Undetermined | 25.84 | Undetermined |
| 30 | Undetermined | 27.23 | Undetermined |
| 31 | Undetermined | 25.69 | Undetermined |
| 32 | Undetermined | 28.83 | Undetermined |
| 33 | Undetermined | 26.65 | Undetermined |
| 34 | Undetermined | 30.32 | Undetermined |
| 35 | Undetermined | Undetermined | 28.44 |
| 36 | 25.36 | 28.42 | Undetermined |
| 37 | 28.63 | 27.25 | Undetermined |
| 38 | 30.28 | 29.69 | Undetermined |
| 39 | 30.12 | 31.44 | Undetermined |
| 40 | 26.65 | 28.56 | Undetermined |
| 41 | 25.42 | 32.37 | Undetermined |
| 42 | 30.13 | 28.28 | Undetermined |
| 43 | 32.28 | 33.56 | Undetermined |
| 44 | 25.63 | Undetermined | 32.76 |
| 45 | 28.52 | Undetermined | 31.35 |
| 46 | 30.26 | Undetermined | 27.35 |
| 47 | 24.65 | Undetermined | 26.38 |
| 48 | Undetermined | 30.58 | 32.45 |
| 49 | Undetermined | 27.51 | 29.68 |
| 50 | 25.63 | 28.36 | 30.32 |
